# Maternal mHealth usability strengthened by trust, governance, and navigation

**DOI:** 10.3389/fdgth.2026.1825544

**Published:** 2026-09-11

**Authors:** Ehiremen Adesua Azugbene

**Affiliations:** 1 College of Health Solutions, Arizona State University, Phoenix, AZ, United States; 2 Maternal and Child Health Innovation Lab, College of Health Solutions, Arizona State University, Phoenix, AZ, United States; 3 Office of Refugee Health, Southwest Interdisciplinary Research Center, Arizona State University, Phoenix, AZ, United States

**Keywords:** digital health governance, health equity, health system alignment, healthcare navigation, maternal health literacy, maternal mHealth, perinatal health, trust in healthcare

## Abstract

Maternal mobile health (mHealth) technologies have expanded rapidly, offering scalable tools to support education, reminders, symptom monitoring, and patient engagement across the perinatal continuum. Despite demonstrated improvements in usability and intermediate behavioral outcomes, persistent disparities in maternal morbidity and mortality highlight the limits of engagement-driven digital innovation. This Perspective advances the viewpoint that maternal mHealth effectiveness depends not only on user-centered design but on alignment with health system structures, governance practices, trust dynamics, healthcare navigation, and navigable care pathways. Drawing on behavioral science, implementation research, digital health governance scholarship, and maternal health equity literature, this paper examines (1) the promise and limits of maternal mHealth, (2) the gap between engagement and clinically meaningful impact, (3) trust as foundational infrastructure, (4) governance and transparency as structural determinants of legitimacy and sustainability, and (5) navigation as the operational bridge between digital activation and real-world maternal healthcare utilization. We propose reframing maternal mHealth as connective infrastructure rather than a feature-driven product model. Within this framework, healthcare navigation is conceptualized as a multidimensional construct comprising behavioral, technological, organizational, and relational dimensions that enable women to move effectively through fragmented healthcare systems. In this framework, usability and engagement remain essential but must be integrated with transparent governance, participatory processes, culturally responsive navigation, and system alignment to translate digital prompts into timely care utilization and equitable maternal health outcomes. Designing for both systems and users positions maternal mHealth as a structural intervention capable of advancing perinatal engagement and reducing disparities.

## 1 Background

**Figure 1:**
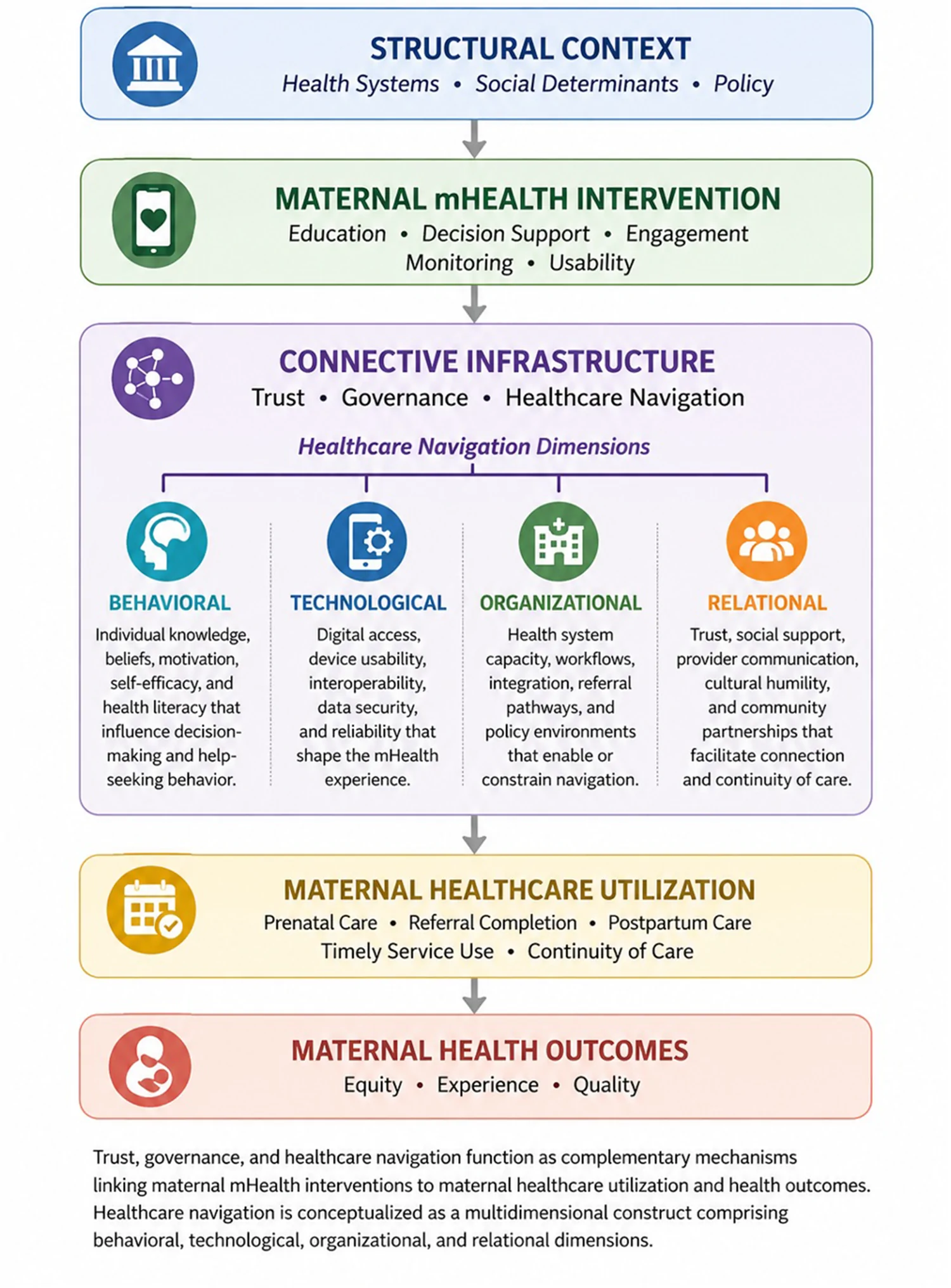
Maternal mHealth as connective infrastructure: A conceptual framework linking digital engagement to maternal healthcare utilization and health outcomes.

Maternal mobile health (mHealth) technologies have expanded rapidly over the past decade, driven by advances in digital innovation and urgent calls to reduce persistent maternal health disparities (1, 2). While many interventions emphasize usability, engagement, and behavioral nudges, inequities in maternal outcomes remain pronounced, particularly among structurally marginalized populations (3–6)*.* Existing maternal mHealth scholarship has increasingly expanded beyond usability and engagement; however, comparatively less attention has been given to how trust, governance, and healthcare navigation collectively function to translate digital engagement into maternal healthcare utilization and equitable maternal health outcomes. *This Perspective advances the position that successful maternal mHealth depends not only on user-centered design but also on alignment with health system structures, governance, trust, and navigable care pathways.* The paper conceptualizes maternal mHealth as connective infrastructure that links women to healthcare systems through coordinated technological, organizational, relational, and governance mechanisms. Drawing on insights from sociotechnical systems thinking and digital health equity scholarship, this perspective recognizes that meaningful improvements in maternal healthcare utilization emerge through interactions among individuals, digital technologies, healthcare systems, and the structural environments in which care is delivered. Within this framework, trust, governance, and healthcare navigation function as complementary system-level mechanisms that enable digital engagement to translate into timely, equitable, and clinically meaningful maternal care. To advance this perspective, we examine (1) the promise and limits of maternal mHealth (2), the gap between engagement and measurable impact (3), trust as foundational infrastructure (4), governance and transparency in digital systems (5), navigation as the bridge between systems and behavior, and (6) a systems-informed model for designing durable maternal mHealth interventions. The conceptual relationships among maternal mHealth, trust, governance, healthcare navigation, and maternal healthcare utilization are summarized in Figure 1.

## 2 The promise and limits of maternal mHealth

Digital innovation has reshaped maternal health care delivery across diverse contexts. Mobile applications now support prenatal education, appointment reminders, symptom tracking, postpartum mental health screening, and lifestyle modification (7–13). Global reviews indicate that mHealth interventions are widely adopted to improve maternal service utilization and patient engagement, particularly in perinatal periods (3, 14, 15). These tools offer scalability, personalization, and real-time communication that traditional care models often lack.

Behavior change frameworks frequently guide maternal mHealth design (10). Reminders, goal-setting prompts, educational modules, and feedback dashboards aim to increase adherence to recommended care practices (8). Evidence suggests that such strategies can improve intermediate outcomes, including appointment attendance and self-monitoring behaviors (1, 3). Digital platforms, therefore, have the potential to strengthen and complement clinical care delivery. Yet persistent disparities in maternal morbidity and mortality reveal the limits of technological optimism (16, 17). Black women in the United States continue to experience disproportionately high rates of maternal complications and death, even after accounting for socioeconomic status and education (16, 18–20). Linguistically diverse and low-income populations encounter fragmented care pathways, inconsistent communication, and structural barriers that digital reminders alone cannot resolve (21). These inequities underscore that access to information does not equate to access to responsive care.

Implementation research demonstrates that digital interventions achieve variable impact depending on system readiness and alignment with real-world care pathways (22, 23). Digital applications deployed without coordination with referral networks, provider workflows, or clear escalation protocols may increase awareness without improving health outcomes (24). Engagement metrics alone cannot determine whether digital prompts translate into timely and appropriate clinical action.

The promise of maternal mHealth lies in its ability to connect individuals to care; its limitation arises when that connection ends at the digital interface rather than extending into coordinated health systems. An effective maternal mHealth strategy must therefore preserve the strengths of behavior change design while addressing the structural conditions that determine whether digital activation results in meaningful health improvement.

## 3 From engagement to impact: the need for system alignment

Maternal mobile health (mHealth) technologies have proliferated in recent years, integrating educational content, automated reminders, and interactive features to engage users throughout pregnancy and the postpartum period (8, 14, 25). Systematic syntheses indicate that these applications are widely deployed to support maternal care delivery, with documented improvements in intermediate outcomes such as service utilization, maternal anxiety reduction, and lifestyle behavior modification during pregnancy (3, 14).

Despite functional improvement in user interfaces and targeted engagement strategies, evidence for consistent improvements in clinically meaningful maternal outcomes remains mixed, particularly where underlying care infrastructures are weak or fragmented (23). Meta-analytic findings suggest that digital health interventions have uncertain effects on key outcomes such as gestational weight gain and maternal-neonatal health when deployed in isolation without system alignment (26).

High engagement rates alone do not guarantee improved health outcomes. Design research examining real-world acceptability and usability indicates that pregnant users may interact positively with digital applications yet continue to face structural barriers when attempting to access follow-up care, interpret clinical guidance, or navigate complex insurance and referral processes (27–29). These findings suggest that usability and engagement, while necessary, are insufficient when digital tools fail to support meaningful interaction with existing care pathways (27–29). Variability in reported maternal mHealth effectiveness suggests that engagement should not be interpreted as a universal indicator of clinical impact (3, 23, 26, 30). Interventions implemented within integrated healthcare systems may achieve greater improvements in care utilization than those introduced into fragmented health systems where referral pathways, provider communication, and organizational readiness remain limited (23, 24, 31, 32). Likewise, interventions emphasizing personalized support may improve patient experience but present challenges for scalability, whereas highly scalable interventions may be lacking in contextual responsiveness (6, 25, 31, 33). Collectively, these findings indicate that differences in maternal mHealth effectiveness reflect not only application design but also the organizational and structural contexts into which these technologies are introduced (3, 22, 23, 26). Maternal mHealth interventions that clarify next steps, map available services, enhance health literacy, and guide users through referral and appointment processes may strengthen healthcare system engagement without requiring direct integration into electronic health record systems. In this context, digital tools function not as replacements for care infrastructure but as navigational bridges that facilitate the timely utilization of available maternal health services.

Evidence also indicates that digital health interventions yield variable results depending on how well they align with existing care pathways and service structures (23). Improvements in interface design or engagement features do not automatically translate into improved outcomes if users encounter fragmented referral systems, unclear next steps, or limited follow-up support (30). When digital prompts encourage action, but health systems remain difficult to navigate, applications may increase awareness without improving care utilization (24). In this context, maternal mHealth must extend beyond behavior change prompts to support health literacy, clarify care pathways, and facilitate timely engagement with available services. Digital tools will function more effectively when they help individuals move through real-world systems that are responsive, coordinated, and accessible.

This Perspective is primarily situated within the United States maternal healthcare context, while recognizing that the foundational principles of trust, governance, healthcare navigation, and health system alignment may have relevance across diverse global settings. Their operationalization, however, is context dependent. In low- and middle-income countries (LMICs), maternal mHealth implementation often occurs within resource-constrained health systems where workforce shortages, infrastructure limitations, and fragmented referral pathways present additional implementation challenges (3, 34–36).

Governance structures, data protections, referral pathways, workforce models, community health worker roles, and access to digital technologies may differ substantially across settings and therefore require adaptation to local healthcare structures, resources, regulatory environments, and population needs. These contextual differences reinforce the importance of designing maternal mHealth interventions that are responsive to local health system capacity while preserving the foundational principles of trust, governance, and navigation (3, 12, 37).

## 4 Trust as foundational infrastructure

Trust is not a peripheral feature of maternal digital health; it is a structural determinant of engagement (38). Persistent disparities in maternal morbidity and mortality among Black, Indigenous, and other women of color (BIPOC) demonstrate how bias and institutional inequity shape the structural context in which digital innovation functions (19, 39). Applications do not exist in isolation from the systems that shape perceptions of safety, legitimacy, and respect (40, 41). When maternal health systems have historically failed certain populations, digital tools inherit that legacy (24, 38). Any mHealth intervention seeking to improve outcomes must therefore contend not only with usability, but with the conditions that produce or undermine trust.

Trust directly shapes whether users disclose symptoms, adhere to guidance, or rely on digital recommendations during vulnerable moments (42). Perceived credibility, transparency, and institutional accountability consistently predict sustained digital engagement (38). Trust within maternal digital health is multidimensional, encompassing interpersonal trust (confidence in healthcare providers and patient-provider relationships), institutional trust (confidence in healthcare organizations and health systems), and technological trust (confidence in the safety, reliability, transparency, and functionality of digital technologies) (38, 42–44). These dimensions are closely interconnected. Trust in digital tools is strengthened when healthcare institutions demonstrate accountability, transparency, and responsiveness, while trustworthy technologies reinforce confidence in the broader healthcare system. Variability in maternal mHealth adoption therefore reflects not only technological performance but also differences in institutional credibility, historical experiences with healthcare systems, and the quality of patient-provider relationships.

Linguistic diversity further complicates this landscape (15, 45). Translation alone does not guarantee comprehension, cultural resonance, or emotional safety (45). Multilingual interfaces that fail to address tone, contextual relevance, or health literacy gaps may increase access while leaving deeper trust deficits intact (46). Transparency in data practices and governance is equally critical. Clear communication about what information is collected, how it is used, and who can access it strengthens user confidence, particularly in contexts where privacy concerns intersect with domestic safety, immigration status, or stigmatized conditions. Participatory feedback mechanisms, in which communities influence iteration and refinement, reinforce legitimacy by signaling that digital tools are accountable to those they serve (47).

Conceptualizing trust as infrastructure rather than sentiment reframes maternal mHealth design priorities. Engagement metrics and download counts provide limited insight into whether users feel safe, heard, or supported (48, 49). Durable impact requires coherence between digital guidance and institutional response (23). Trust is reinforced when recommendations are matched by accessible services, responsive providers, and navigable care pathways (22, 38). Erosion of trust occurs when digital prompts exceed system capacity or fail to acknowledge lived realities. Positioning trust as foundational shifts maternal mHealth from a feature-driven model toward a system-aligned approach in which relational credibility and structural responsiveness determine sustained engagement and meaningful health improvement.

## 5 Governance and transparency in digital maternal health

Governance determines whether maternal mHealth tools are perceived as legitimate, accountable, and safe. While usability and navigation shape user experience, governance defines the structural conditions under which digital systems operate. Contemporary digital health scholarship underscores that data stewardship, transparency, and institutional accountability are central to sustained adoption and long-term credibility (43). Maternal mHealth platforms function within this broader governance landscape, where clarity regarding data practices, oversight, and responsibility directly influences both user trust and provider engagement (38, 42).

Data transparency is particularly consequential in maternal health contexts. Applications routinely collect sensitive information related to pregnancy status, mental health, reproductive decision-making, and social circumstances (50). Users are more likely to engage consistently when they understand what information is collected, how it is used, and who can access it (38, 51). Privacy protections and clearly articulated accountability structures reduce uncertainty and reinforce perceptions of safety (50, 52). Governance, however, extends beyond data management. Decision-making processes influence how digital tools evolve, whose voices shape iteration, and whether design priorities reflect lived realities (38, 52). Structured stakeholder engagement, including patients, clinicians, and community representatives, strengthens alignment with care pathways and enhances sustainability (6, 33, 51). Participatory co-design processes ensure that governance structures, navigation tools, and trust-building mechanisms reflect the lived experiences of the populations they are intended to serve (53). Inclusive governance does not require diffuse ownership; it requires transparent mechanisms for participation, feedback, and accountability.

Governance also requires balancing competing implementation priorities. Strong governance structures promote transparency, accountability, patient safety, and public trust; however, they may also introduce additional organizational complexity, implementation burden, and slower innovation when oversight processes become overly rigid (31, 43). Conversely, governance models that prioritize rapid deployment without adequate oversight may increase risks related to inconsistent privacy protections, inequitable implementation, and diminish public confidence (50, 52). Sustainable maternal mHealth implementation therefore depends not only on effective governance, but on governance that appropriately balances innovation, accountability, equity, and organizational feasibility across diverse healthcare settings.

Institutional alignment determines whether digital maternal health tools operate as isolated applications or as coordinated supports within broader care systems (23). Governance structures that define referral expectations, response timelines, and escalation pathways ensure that digital prompts correspond to accessible and actionable services (31, 32). Without this clarity, digital tools may increase awareness yet fail to convert engagement into maternal healthcare utilization. Successful implementation depends on addressing practical health system barriers that extend beyond application design, including interoperability with existing clinical systems, workforce capacity, digital infrastructure, reimbursement mechanisms, and organizational readiness (23, 24, 31, 32, 43). Collectively, these implementation realities demonstrate that sustainable maternal mHealth requires coordinated health system investment alongside technological innovation to achieve long-term adoption and meaningful maternal healthcare impact (6, 10, 33).

Positioning governance as a foundation can transform maternal mHealth from a feature-driven product model toward a system-aligned intervention framework. Privacy safeguards, participatory processes, and clearly articulated accountability structures are not peripheral design elements; they are structural determinants of equity, legitimacy, and sustained impact. Governance must also anticipate potential digital harms, including algorithmic bias, reproductive data misuse, misinformation amplification, digital exclusion, and surveillance concerns that may disproportionately affect historically underserved populations (42, 50, 52). As artificial intelligence becomes increasingly integrated into maternal mHealth applications, governance frameworks should also ensure that AI-enabled decision support is transparent, clinically appropriate, accountable, and equitable (42, 52). Addressing risks through transparent governance, ethical oversight, and equitable implementation is essential to ensure that maternal mHealth technologies promote trust while minimizing unintended harm.

## 6 Navigation as the bridge between systems and behavior

Navigation functions as the operational link between digital engagement and real-world maternal healthcare utilization. Many maternal mHealth applications emphasize reminders, educational content, and behavior prompts to encourage attendance and self-monitoring (8). While these features can increase awareness, women still must identify appropriate services, determine which providers meet their needs, and understand how to move through complex health systems. Without clear guidance on where to go, whom to contact, and how services differ, digital activation may stall before meaningful care engagement occurs (23). This gap between awareness and action reflects a broader limitation in maternal health literacy, where knowledge alone does not guarantee the ability to navigate care systems effectively (15, 60).

Building on existing healthcare and patient navigation literature (54–57), healthcare navigation is conceptualized in this Perspective as a multidimensional construct comprising behavioral, technological, organizational, and relational dimensions that collectively enable individuals to identify, access, coordinate, and move through fragmented healthcare systems to obtain timely and appropriate care. Within the maternal health context, maternal healthcare navigation extends beyond understanding health information by supporting movement through prenatal, intrapartum, and postpartum care pathways using digital tools, healthcare organizations, and trusted interpersonal support. Behavioral navigation encompasses the actions individuals take to identify care needs, seek services, schedule and attend care, and follow through with recommended care. Technological navigation refers to the use of digital tools to locate services, obtain guidance, manage appointments, and facilitate connections to care. Organizational navigation involves moving through healthcare structures and processes, including eligibility requirements, insurance systems, referrals, and care pathways. Relational navigation reflects support from trusted individuals—including patient navigators, community health workers, doulas, clinicians, peers, and family members—who help individuals understand and move through healthcare systems. As conceptualized in this Perspective, maternal healthcare navigation is distinct from maternal health literacy, which focuses on understanding and applying health information; patient engagement, which emphasizes active participation in care; care coordination, which focuses on organizing services across providers; and referral facilitation, which focuses on connecting individuals to appropriate services. Rather than replacing these constructs, healthcare navigation integrates them into a system-oriented process that enables women to successfully move through maternal healthcare systems.

Maternal health literacy extends beyond knowledge of symptoms or guidelines (15, 60). The concept includes the ability to locate culturally responsive providers, access services that align with insurance coverage and geographic proximity, and identify supports such as doulas, community health workers, or peer networks (58, 60). Navigation tools that curate trusted resources, clarify eligibility requirements, and offer direct linkage to relevant services reduce structural friction (23, 59). Clear mapping of care options in ways that are tailored, linguistically appropriate, and context-sensitive enables digital platforms to help women connect to care pathways that reflect their lived realities rather than forcing them to navigate fragmented systems independently (14).

Effective navigation does not replace healthcare systems; it strengthens engagement with them (23). Linkage mechanisms that guide users toward appropriate clinics, community-based supports, and responsive providers translate digital prompts into real-world action (23). When digital tools connect women to services that are accessible, culturally concordant, and geographically feasible, engagement evolves into utilization (23, 37). Navigation, therefore, operates as connective infrastructure, aligning individual behavior with available care systems and enabling timely, coordinated maternal health support.

## 7 Conclusion

Usability and engagement remain essential foundations of maternal mHealth design. However, equitable and durable impact requires extending these strengths beyond the digital interface into the structures that shape real-world care. Evidence from behavioral science, implementation research, and maternal health equity scholarship demonstrates that meaningful improvement emerges when digital tools align with navigable care pathways, transparent governance practices, and institutional responsiveness. Trust, governance, and navigation function not as alternatives to usability, but as complementary conditions that enable engagement to translate into maternal healthcare utilization and improved outcomes.

Reframing maternal mHealth as connective infrastructure expands the focus from downloads and interaction metrics toward coordinated system participation. Digital platforms can enhance maternal health literacy, clarify next steps, and facilitate timely linkage to culturally responsive services when they are designed to support movement through existing care systems. Alignment between digital guidance and service availability determines whether technology strengthens engagement or merely increases awareness. Designing for both systems and users positions maternal mHealth as a structural intervention capable of advancing perinatal engagement and reducing disparities.

Future research should evaluate how trust, governance, and healthcare navigation function as complementary mechanisms influencing maternal healthcare utilization across diverse health system contexts. Implementation efforts should prioritize health system integration, equitable digital infrastructure, and sustainable workforce support, while policy development should establish governance frameworks that promote transparency, accountability, and equitable access to maternal digital health technologies.

When usability, behavioral design, governance, trust, and navigation operate in concert, digital innovation becomes more than a communication tool; it becomes an enabling infrastructure for equitable maternal health impact.
